# Association of excessive daytime sleepiness with migraine and headache frequency in the general population

**DOI:** 10.1186/s10194-017-0743-0

**Published:** 2017-03-20

**Authors:** Knut Stavem, Håvard Anton Kristiansen, Espen Saxhaug Kristoffersen, Kari Jorunn Kværner, Michael Bjørn Russell

**Affiliations:** 1 Head and Neck Research Group, Research Centre, Akershus University Hospital, Lørenskog, Norway; 2 Institute of Clinical Medicine, Campus Akershus University Hospital, University of Oslo, Lørenskog, Norway; 3 Department of Pulmonary Medicine, Medical Division, Akershus University Hospital, Lørenskog, Norway; 4 Health Services Research Unit, Akershus University Hospital, Lørenskog, Norway; 5 Department of General Practice, University of Oslo, Oslo, Norway; 6 C3 Centre for Connected Care, Oslo University Hospital, Oslo, Norway; 7 BI Norwegian Business School, Oslo, Norway

**Keywords:** Epidemiology, Excessive daytime sleepiness, Headache, Migraine

## Abstract

**Background:**

Some previous studies have postulated an association between migraine and excessive daytime sleepiness (EDS). This study evaluated the association of EDS with migraine and headache frequency in a general population, after adjusting for potential confounding variables.

**Methods:**

The study was a postal survey of a random age and gender-stratified sample of 40,000 persons aged 20 to 80 years old drawn by the National Population Register in Norway. The questionnaire included questions about migraine, headache, the Epworth sleepiness scale (ESS) and various comorbidities. EDS was defined as ESS > 10. The association of EDS and migraine/headache were analysed by bivariate and multivariable logistic regression analyses.

**Results:**

A total of 21,177 persons responded to the ESS and were included in the analyses. The odds ratio (OR) for EDS was increased for migraineurs (1.42 (95% CI 1.31─1.54), *p <* 0.001) compared to non-migraineurs; however, this finding was not significant after adjustment for a number of possible confounders. EDS increased with increasing headache frequency, with an OR of 2.74 (95% CI 2.05─3.65), *p <* 0.001) for those with headache on >179 days per year compared to those without headache in multivariable analysis.

**Conclusions:**

In a general population, the odds for EDS increased significantly with the headache frequency, irrespective of migraine status. EDS was not associated with reported migraine in multivariable analysis.

## Background

Headache is the most common type of pain experienced by man, but receives little attention, possibly due to the often mild symptoms, and because most people rarely consult their physician due to headache [1]. Almost everyone experience tension-type headache once in a while, and migraine affects 10–20% of the general population [2, 3]. About 3% of the world population have chronic headache, i.e. mainly chronic tension-type headache or chronic migraine [4, 5].

Persons with headache and/or migraine often complain of sleepiness, a symptom with high clinical and public health importance due to increased risk for accidents, decreased productivity and impaired quality of life [6–9]. Excessive daytime sleepiness (EDS) is experienced by 8─30% of the general population [10–13]. Despite the considerable burden of EDS, it is still under-reported, under-diagnosed and under-treated among those with chronic pain as well as in the general population [14–16].

Some clinic-based [17–20] or population-based [21] studies have proposed an association between migraine and EDS. Recently a large population-based study reported an association between migraine and EDS in bivariate analysis, but this association was weakened in multivariable analysis and became non-significant when adjusting for anxiety, depression, sleep duration and a score for sleep quality [22]. Moreover, whether EDS is associated with headache frequency in migraineurs varies between studies [21, 22].

The present study aimed to investigate the association of EDS with migraine and headache frequency in an age and gender-stratified general population sample, after adjusting for possible confounders.

## Methods

### Study design and sample

An age and gender-stratified random sample of 40,000 persons aged 20─80 years old were drawn by the National Population Register of Statistics Norway. Each of the ages 30, 35, 40, 45, 50, 55 and 60 years included 2,000 persons of each gender, while the remaining ages included 1,000 persons of each gender. The participants were residing in Akershus, Hedmark or Oppland County. The sample size was reduced to 38,871 because of error in the address list (*n =* 1,024), multi-handicap (*n =* 4), dementia (*n =* 23), insufficient Norwegian language skills (*n =* 3) and deceased (*n =* 75). The three counties have both rural and urban areas, and Akershus County is situated in close proximity to Oslo. Details of the study have previously been presented [23].

### Postal survey

All participants received a mailed questionnaire with a standard letter containing information about the project. Apart from ensuring confidentiality and emphasising the importance of participation, it was stated that the objective was to study sleepiness during daytime. If the questionnaire evoked no response, a second mail was issued. The replies could either be on paper or web-based. The data were collected between February and August 2006. All questionnaires were scanned using TeleForm version 9 (Cardiff Software Ltd, Cambridge, UK).

### Migraine and headache

The questions: “How many days during the last year have you had headache? (0 days, 1─11 days, 12─30 days, 31─84 days, 85─179 days or ≥180 days)”, and “Have you ever had migraine? (yes or no)” were used to screen for headache frequency and migraine. We applied the International Classification of Headache Disorders (ICHD-II) definitions slightly modified, i.e. no headache is 0 headache days within the last year, infrequent headache is 1–11 headache days within the last year, frequent headache is 12─179 headache days within the last year, while chronic headache is 180 days or more within the last year [24]. The term non-migraineurs describes those without migraine, independent of whether they reported headache or not.

### Daytime sleepiness and average sleep time

Daytime sleepiness was assessed by the Epworth Sleepiness Scale (ESS). The ESS is a standardized questionnaire which describes eight daily situations in which the respondents estimate their likelihood of dozing off on a scale of 0 to 3, i.e. 0 = no chance of dozing, 1 = slight chance of dozing, 2 = moderate chance of dozing and 3 = high chance of dozing [25]. The ESS score thereby ranges from 0 to 24, and the results were dichotomized into scores ≤10 and >10, the latter is considered to represent clinically significant EDS [26]. A question of average sleep time duration during the night was answered in hours and minutes and was categorized into three classes: short (<6 h), normal (6─8 h) and long (>8 h) sleep duration. In Norway, the ESS has exhibited properties in line with other language versions of the questionnaire [27].

### Smoking status and body mass index

Smoking status was assessed with the item “How many cigarettes do you usually smoke per day?”, coded as none, 1─10, 11─20, >20 for the analyses. Body mass index (BMI) in kg/m^2^ was calculated from self-reported weight and height.

### Comorbidity

To screen for depression, we asked the question: “Have you felt depressed or experienced diminished interest or pleasure in activities you usually enjoy?” This is a combination of two questions, where a “yes” answer to either of the two questions was considered a positive test [28]. The replies were dichotomized, i.e. never or rarely, 1─2 times a month, 1─2 times a week or 3─4 times a week were classified as no depression, while almost daily was classified as depression.

The participants also responded to items about other comorbidities: previous myocardial infarction (yes or no), stroke (yes or no), current/previous angina pectoris (yes or no), asthma (yes or no), allergy (yes or no), hypertension (yes, no or unknown), or diabetes (yes or no). Finally, they responded to an item about ever having had treatment for a sleep disorder (yes or no).

### Statistical analyses

Descriptive statistics are presented using the mean (SD) or number (%). Groups were compared using the chi-square test.

Prevalence estimates are presented with cross-tables. To assess possible effect modification, the homogeneity of odds ratios (OR) for EDS in migraine vs. non-migraine was tested in stratified analyses using the chi-square test.

The association of EDS with migraine and headache frequency was analysed by bivariate and multivariable logistic regression analyses with EDS (ESS > 10 = 1, ESS ≤ 10 = 0) as the dependent variable. The six independent variables migraine, headache frequency, age, gender, BMI, and depression were included in the model. The modelling was conducted in several steps: (1) Bivariate models, (2) Multivariable models with the six variables mentioned above, (3) Multivariable models as (2) with the addition of the variables of allergy, angina pectoris, asthma, diabetes, hypertension, myocardial infarction and stroke, and (4) Multivariable models as (3) with the addition of the variables cigarette smoking, nightly sleep duration, and treatment for sleep disorder. Missing values were not imputed, and deletion of cases in the models was listwise.

Tests for multiplicative interactions were conducted between migraine*gender, headache frequency*gender, and migraine*headache frequency. Finally, model (3) was replicated in subsets of the respondents: non-migraineurs, migraineurs, men, and women.

All statistical analyses were performed using Stata version 14.1 (StataCorp, College Station, TX). We chose a 5% significance level, using two-sided tests.

### Ethical issues

The project was approved by The Regional Committees for Medical Research Ethics and the Norwegian Social Science Data Services.

## Results

The overall response rate was 54.5% (21,177/38,871). The respondents had a mean (SD) age of 50.5 (15.6) years and comprised 52.5% women (Table 1). Complete information on headache and migraine was reported by 89.4% of women and by 88.0% of men. Regarding replies to the ESS, 4.7% of women and 3.3% of men had missing values in one question, 0.7% of women and 0.6% of men had missing values in two questions, while 1.2% of women and 0.7% of men had missing values in three or more questions.

**Table 1:** Descriptive statistics for survey respondents, number (%) unless otherwise stated

	*N*	
Age in years, mean (SD)	21,177	50.5 (15.6)
Gender, women	21,177	11,120 (53)
Epworth sleepiness scale score, mean (SD)	21,177	6.7 (4.0)
Epworth sleepiness scale score >10	21,177	3,501 (17)
Migraine	19,833	5,258 (27)
Headache frequency (days last year)	20,021	
None		4,559 (23)
1-11		8,109 (41)
12-30		3,547 (18)
31-84		2,280 (11)
85-179		951 (5)
180 days or more		575 (3)
Body mass index in kg/m^2^, mean (SD)	20,818	25.8 (4.2)
Comorbidity		
Symptoms of depression	20,476	1,401 (7)
Myocardial infarction	20,686	814 (4)
Stroke	19,938	536 (3)
Angina pectoris	20,681	978 (5)
Hypertension	20,868	
No		14,903 (71)
Yes		3,591 (17)
Unkown		2,374 (11)
Diabetes	20,685	934 (5)
Allergy	20,121	6,564 (33)
Asthma	19,156	2,287 (12)
Treated for sleep disorder	20,749	1,251 (6)
Smoking (cigarettes per day)	17,286	
None		11,902 (69)
0─10		2,867 (17)
11─20		2,252 (13)
>20		265 (2)
Average nightly hours slept, mean (SD)	20,907	7.0 (1.1)
Average nightly hours slept, categorized		
<6		1,750 (8)
6-8		17,541 (83)
>8		1,886 (9)

**Fig. 1:**
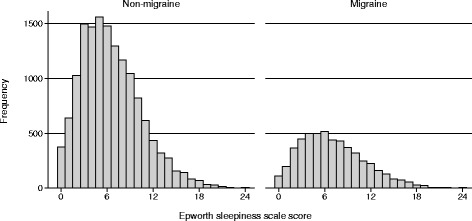
Histogram of the frequency of Epworth sleepiness scale scores for participants according to migraine or non-migraine status

**Fig. 2:**
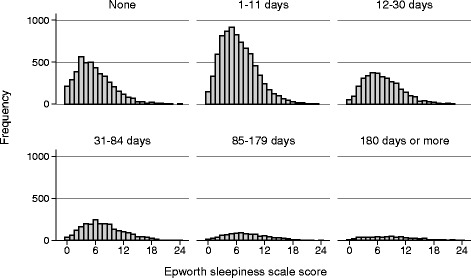
Histogram of the frequency of Epworth sleepiness scale scores for participants according to categories of headache frequency (in number of days per month)

The 1-year prevalence of headache was 77.2%; 84.0% (8,872/10,558) in women and 69.6% (6,590/9,463) in men. The lifetime prevalence of migraine was 26.5%; 34.1% (3,562/10,441) in women and 18.1% (1,696/9,392) in men (*p <* 0.001). In the total population the mean (SD) ESS score was 6.7 (4.0); among migraineurs 7.1 (4.2) (*n* = 5,258) and non-migraineurs 6.5 (3.9) (*n* = 14,575). The ESS scores according to migraine status (Fig. 1) and headache frequency (Fig. 2) showed skewed distributions in most subgroups.

Among migraineurs, non-migraineurs, and those with a missing response to the migraine item, the prevalence of EDS increased with headache frequency in all three groups (Table 2). The overall prevalence of EDS among the respondents (*n* = 21,177) was 17%, and it was 20% among migraineurs, 15% among non-migraineurs, and 19% among those with missing value on the migraine item.

**Table 2:** Prevalence of excessive daytime sleepiness (EDS), defined as Epworth sleepiness scale score >10, according to migraine and headache frequency

	Non-migraine		Migraine		Missing migraine item		Total	
Headache frequency, days last year	*N*	*n (%)*	*N*	*n (%)*	*N*	*n (%)*	*N*	*n (%)*
None	3,814	413 (11)	390	56 (14)	355	56 (16)	4,559	525 (12)
1–11	6,159	862 (14)	1,568	228 (15)	382	59 (15)	8,109	1,149 (14)
12–30	2,040	378 (19)	1,302	238 (18)	205	41 (20)	3,547	657 (19)
31–84	1,019	227 (22)	1,087	271 (25)	174	42 (24)	2,280	540 (24)
85–179	411	115 (28)	482	139 (29)	58	14 (24)	951	268 (28)
≥180	219	76 (35)	294	102 (35)	62	22 (35)	575	200 (35)
Missing	913	124 (14)	135	22 (16)	108	15 (14)	1,156	162 (14)
Total	14,575	2,196 (15)	5,258	1,056 (20)	1,236	234 (19)	21,177	3,501 (17)

There was no indication of heterogeneity of the ORs for EDS in migraine vs. non-migraine, or women vs. men in strata according to headache frequency (Table 3).

**Table 3:** Odds ratios and 95% confidence intervals (95% CI) for having excessive daytime sleepiness (EDS) for migraineurs compared to non-migraineurs, and men versus women. Stratified analyses according to headache frequency categories

	Migraine vs. non-migraine		Men vs. women	
Headache frequency, days last year	Odds ratio (95% CI)	*P*	Odds ratio (95% CI)	*P*
None	1.38 (1.02─1.87)	0.035	1.25 (1.03─1.51)	0.026
1─11	1.05 (0.89─1.22)	0.58	1.35 (1.19─1.53)	<0.001
12─30	0.98 (0.82─1.18)	0.86	1.34 (1.13─1.59)	<0.001
31─84	1.16 (0.95─1.42)	0.152	1.36 (1.11─1.66)	0.003
85─179	1.04 (0.78─1.40)	0.78	1.21 (0.90─1.64)	0.21
≥180	1.00 (0.69─1.44)	1.00	0.91 (0.63─1.32)	0.62
Missing	1.23 (0.75─2.01)	0.42	1.37 (0.98─1.91)	0.068
Test of homogeneity of odds ratios:	Chi^2^(6) = 4.69, *P* = 0.58		Chi^2^(6) = 4.73, *P* = 0.58	

In bivariate logistic regression analysis, the odds ratio (OR) for having EDS was higher among migraineurs than non-migraineurs, and the OR for EDS increased with increasing headache frequency (Table 4).

**Table 4:** Odds ratios with 95% confidence intervals (95%CI) for having excessive daytime sleepiness (EDS), logistic regression analysis

	Bivariate analysis		Multivariable analysis		Multivariable analysis^a^		Multivariable analysis^b^	
*N*	19,833─21,177		18,027		15,299		12,369	
	Odds ratio (95%CI)	*P*	Odds ratio (95%CI)	*P*	Odds ratio (95%CI)	*P*	Odds ratio (95%CI)	*P*
Migraine								
No	1		1					
Yes	1.42 (1.31─1.54)	<0.001	1.11 (1.01─1.22)	0.032	1.11 (1.00─1.23)	0.044	1.08 (0.96─1.21)	0.196
Headache frequency, days per year								
None	1		1		1		1	
1─11	1.27 (1.14─1.42)	<0.001	1.18 (1.05─1.34)	0.009	1.23 (1.07─1.41)	0.003	1.17 (1.00─1.36)	0.05
12─30	1.75 (1.54─1.98)	<0.001	1.54 (1.33─1.78)	<0.001	1.53 (1.31─1.80)	<0.001	1.50 (1.25─1.79)	<0.001
31─84	2.38 (2.09─2.72)	<0.001	2.07 (1.77─2.42)	<0.001	2.02 (1.70─2.40)	<0.001	2.00 (1.65─2.43)	<0.001
85─179	3.02 (2.55─3.57)	<0.001	2.53 (2.08─3.06)	<0.001	2.39 (1.93─2.96)	<0.001	2.26 (1.77─2.87)	<0.001
≥180	4.10 (3.37─4.98)	<0.001	3.12 (2.49─3.92)	<0.001	3.12 (2.42─4.02)	<0.001	2.74 (2.05─3.65)	<0.001
Age, per 10 years	0.85 (0.83─0.87)	<0.001	0.87 (0.85─0.90)	<0.001	0.88 (0.85─0.91)	<0.001	0.88 (0.84─0.91)	<0.001
Gender								
Women	1		1		1		1	
Men	1.10 (1.02─1.18)	0.01	1.26 (1.16─1.37)	<0.001	1.21 (1.11─1.33)	<0.001	1.22 (1.10─1.35)	<0.001
Body mass index, per kg/m2	1.05 (1.04─1.06)	<0.001	1.05 (1.04─1.06)	<0.001	1.04 (1.03─1.05)	<0.001	1.04 (1.02─1.05)	<0.001
Symptoms of depression								
No	1		1		1		1	
Yes	2.99 (2.66─3.36)	<0.001	2.45 (2.15─2.80)	<0.001	2.48 (2.13─2.88)	<0.001	2.20 (1.86─2.62)	<0.001

^a^adjusted for additional potential confounders allergy, angina pectoris, asthma, diabetes, hypertension, myocardial infarction and stroke

^b^adjusted for additional potential confounders (as above) + cigarette smoking (0, 1─10, 11─20, >20 per day), nightly sleep (<6 h, 6─8 h, >8 h) and previous treatment for sleep disorder

In multivariable logistic regression analysis, the relationship between migraine and EDS was weaker than in bivariate analysis, while the pattern of increasing OR for EDS with increasing headache frequency persisted in models after adjusting for age, BMI, depression, and gender (Table 4). Adjusting for additional potential comorbidities in two steps did not change this pattern (Table 4). Interaction terms between migraine*gender, headache frequency*gender, and migraine*headache were statistically not significant and were left out in the models.

In multivariable analysis in strata of the respondents, the pattern of ORs was consistent across migraineurs and non-migraineurs after adjusting for headache frequency, age, BMI, depression, gender and other comorbidities, and across women and men after adjusting for migraine, headache frequency, age, BMI, depression and other comorbidities (Table 5).

**Table 5:** Odds ratios and 95% confidence intervals (95% CI) for having excessive daytime sleepiness (EDS), multivariable logistic regression analysis^a^ in strata of the population

	Migraineurs		Non-migraineurs		Men		Women	
*N*	4,126		11,173		7,313		7,986	
	Odds ratio (95%CI)	*P*	Odds ratio (95%CI)	*P*	Odds ratio (95%CI)	*P*	Odds ratio (95%CI)	*P*
Migraine								
No	-		-		1		1	
Yes	-		-		1.18 (1.01─1.38)	0.041	1.07 (0.94─1.22)	0.31
Headache frequency, days per year								
None	1		1		1			
1─11	0.93 (0.63─1.37)	0.73	1.27 (1.09─1.47)	0.002	1.32 (1.11─1.57)	0.001	1.09 (0.86─1.37)	0.48
12─30	1.21 (0.82─1.79)	0.34	1.58 (1.32─1.89)	<0.001	1.63 (1.31─2.03)	<0.001	1.37 (1.07─1.75)	0.013
31─84	1.74 (1.17─2.57)	0.006	1.92 (1.55─2.37)	<0.001	2.30 (1.79─2.94)	<0.001	1.74 (1.34─2.25)	<0.001
85─179	1.90 (1.24─2.91)	0.003	2.52 (1.91─3.34)	<0.001	2.33 (1.65─3.30)	<0.001	2.21 (1.64─2.96)	<0.001
≥180	2.72 (1.71─4.30)	<0.001	2.92 (2.04─4.19)	<0.001	2.43 (1.57─3.77)	<0.001	3.21 (2.30─4.50)	<0.001
Age, per 10 years	0.90 (0.84─0.95)	0.001	0.87 (0.84─0.91)	<0.001	0.92 (0.88─0.97)	0.001	0.85 (0.81─0.89)	<0.001
Sex								
Women	1		1		-		-	
Men	1.31 (1.11─1.54)	0.002	1.18 (1.06─1.32)	0.003	-		-	
Body mass index, per kg/m^2^	1.04 (1.02─1.05)	<0.001	1.04 (1.03─1.06)	<0.001	1.05 (1.03─1.06)	<0.001	1.04 (1.03─1.05)	<0.001
Symptoms of depression								
No	1		1		1			
Yes	2.18 (1.70─2.79)	<0.001	2.66 (2.21─3.21)	<0.001	2.57 (2.05─3.22)	<0.001	2.43 (1.99─2.97)	<0.001

^a^adjusted for additional potential confounders allergy, angina pectoris, asthma, diabetes, hypertension, myocardial infarction and stroke

## Discussion

This population-based cross-sectional study has demonstrated (1) a relationship between migraine and EDS in bivariate analysis, although this association was weakened and eventually disappeared in multivariable analysis, and (2) an association between headache frequency and EDS, which was consistent after adjustment for numerous potential confounders, and also when the analysis was replicated in subsets of the study population, i.e. among migraineurs, non-migraineurs, women or men.

Overall 17% met the criteria for having EDS, which is in line with that of a previous Norwegian telephone survey of a general population [29]. Our 20% prevalence of EDS in migraineurs was identical to that of Korean migraineurs from the general population [22]. The EDS prevalence in European clinic populations was 14% in those with episodic migraine [18] and 20% in chronic migraine [20], while it was substantial higher in an American clinic population, i.e. 32% in episodic migraineurs and 40% in chronic migraineurs [17]. The higher prevalence in the American study may partly be explained by a slightly higher mean BMI and lower ESS cut off, i.e. ≥ 10 rather than >10 in the present and Korean studies. The European clinic studies included 100 person with episodic and chronic migraine, respectively, while the American clinic study included 72 persons with episodic migraine and 128 persons with chronic migraine, providing relative broad confidence intervals for the EDS prevalence, i.e. 14% (8–22%), 20% (13–29%), 32% (25–48%) and 40% (31–49%).

The present study found an association between migraine and EDS in bivariate analysis, which was weaker in the multivariable analyses and disappeared when adjusting for all potential confounders, a finding similar to that of The Korean population-based study when adjusting for anxiety, depression, sleep duration and Pittsburgh Sleep Quality index score [22]. These findings are in concert with another population-based study reporting increased odds of EDS in migraineurs compared with headache-free individuals after adjustment for age, sex and sleep-promoting medication [21]. The findings are also supported by a case–control study of episodic migraine [18, 20], as well as a study including 370 migraineurs and 119 non-migraineurs [20]. The latter study found a statistically significant association of chronic migraine and EDS with an OR of 3.9 (95% CI 1.5.–10.2), although the mean ESS score was not significant different between those with chronic migraine and healthy controls [20].

In contrast, one study reported a statistically significant association between migraine and sleep problems after adjusting for both lifetime and current anxiety and mood disorders [30]. This was a controlled study using a systematic assessment of migraine, sleep problems and anxiety and mood disorders with interviews, in contrast to the present, larger questionnaire-based survey.

In the present study, the prevalence of EDS increased with headache frequency, independent of migraine status, in line with a previous general population study [21], although in the latter study the increased ORs for EDS with increasing headache frequency were statistically not significant. Therefore, it is possibly the frequency of the pain rather than migraine or tension-type headache in itself that is associated with EDS.

There was no evidence of effect modification of gender or headache frequency on the association of migraine with EDS, or of gender on the association of headache frequency with EDS, as indicated by the homogeneity of the ORs and the lack of significant interactions in the regression models.

This study had several strengths. Firstly, it was a very large population-based survey. Secondly, the multivariable analyses were conducted in three consecutive steps and adjusted for an increasing number of possible confounders in each step.

Several potential limitations of this study should be mentioned. The response rate was relatively low; however, it was comparable to that of some other sleep-related epidemiologic studies [29, 31] and higher than in recent population-based studies of headache and sleep [21, 22].

The present study defined EDS as ESS > 10, in line with the recommendation of the developer of the ESS [32], recent epidemiological studies [33–35] and a recent population-based study of headache and sleep [22]. In contrast, for unknown reasons, most previous studies of the association of migraine or headache frequency with EDS defined EDS as ESS ≥10 [17–21], which would lead to a higher prevalence of EDS. The ESS is widely used in evaluating subjective daytime sleepiness [25, 36]. However, the ESS has been criticized because it shows little association with objective measures, such as the multiple sleep latency test (MSLT) [37–39]. The large number of participants in this study precluded the use of MSLT for more comprehensive assessment of daytime sleepiness.

Another potential limitation is that the data reported in the present study were self-reported. This study asked about lifetime prevalence of migraine. The single question about lifetime occurrence of migraine, which was used, has previously been evaluated against a clinical interview by two physicians in another Norwegian survey in eastern Akershus County, reporting a raw agreement rate of 0.81 and kappa (*κ)* of 0.62, which is considered to be a good agreement [23]. Two previous epidemiological surveys used the same screening question for migraine as in the present study, and the observed agreement rates were 0.92 and 0.94, and *κ* were 0.77 and 0.81, respectively [2, 40]. Due to the high number of participants in our survey, it was not feasible to apply the gold standard, i.e. a clinical interview by a physician/neurologist with expertise in headache diagnostics. The current study did not investigate the effect of EDS on clinical characteristics of migraine, which would require more detailed clinical information than is possible in a questionnaire-based general population survey.

Single questions about tension-type headache and its frequency have been compared with a clinical interview, and the agreement was good [3, 40]. We think our question regarding headache was at least as precise as the single question about tension-type headache, because the term headache is known by everybody, while some subjects may not know what tension-type headache is. Similarly, we think the frequency report on headache is likely to be precise.

The data on headache frequency was based on headache during the last year, which is likely to be less subject to recall bias as compared to headache earlier in life.

Moreover, the single question about self-reported depression might be less precise than a more extensive questionnaire or diagnostic interview. However, the combination of the two questions into one item has been shown to have a sensitivity of 96% (95% CI 90─99%) and specificity of 57% (95%CI 53─62%) in relation to major depression [28]. This compares with a sensitivity of 89─96% and specificity 51─72%, when ascertained with more extensive questionnaires [28].

The present study did not assess the quality of sleep, or the use of analgesics that may be associated with headaches and EDS, or β-blockers that may be used in prophylactic treatment of migraine and may be associated with EDS. However, only a small minority of migraineurs use β-blockers.

The focus of this paper was on the association of migraine and headache frequency with EDS, and not on a general prediction model for EDS. Therefore, we have focused on factors that may confound the association of migraine and/or headache frequency with EDS, and on possible effect modification between some of these variables. EDS depends on several factors known to interfere with the sleep-awake cycle, while headache and migraine are mainly known to vary with gender. For example, caffeine, alcohol, employment including nightshifts, restless legs syndrome, concomitant urological diseases, menstruation and thyroid hormones may be associated with EDS. However, based on the literature, we are not aware of firm evidence for an association of these factors with migraine or tension-type headache, and if such an association exists it is most likely of limited importance.

## Conclusions

This study showed that EDS was associated with an increase in headache frequency in both migraineurs and non-migraineurs. In contrast, the association between migraine and EDS disappeared after adjustment for a number of confounders.

## Abbreviations

EDS: Excessive daytime sleepiness
ESS: Epworth sleepiness scale
ICHD: International classification of headache disorders
MSLT: Multiple sleep latency test
SD: Standard deviation

## References

[1] Lyngberg AC, Rasmussen BK, Jorgensen T, Jensen R (2005). Secular changes in health care utilization and work absence for migraine and tension-type headache: a population based study. Eur J Epidemiol.

[2] Russell MB, Rasmussen BK, Thorvaldsen P, Olesen J (1995). Prevalence and sex-ratio of the subtypes of migraine. Int J Epidemiol.

[3] Russell MB, Levi N, Saltyte-Benth J, Fenger K (2006). Tension-type headache in adolescents and adults: a population based study of 33,764 twins. Eur J Epidemiol.

[4] Jensen R, Stovner LJ (2008). Epidemiology and comorbidity of headache. Lancet Neurol.

[5] Grande RB, Aaseth K, Gulbrandsen P, Lundqvist C, Russell MB (2008). Prevalence of primary chronic headache in a population-based sample of 30- to 44-year-old persons. The Akershus study of chronic headache. Neuroepidemiology.

[6] Mitler MM, Carskadon MA, Czeisler CA, Dement WC, Dinges DF, Graeber RC (1988). Catastrophes, sleep, and public policy: consensus report. Sleep.

[7] Roth T, Roehrs TA (1996). Etiologies and sequelae of excessive daytime sleepiness. Clin Ther.

[8] Leger D (1994). The cost of sleep-related accidents: a report for the national commission on sleep disorders research. Sleep.

[9] Briones B, Adams N, Strauss M, Rosenberg C, Whalen C, Carskadon M, Roebuck T, Winters M, Redline S (1996). Relationship between sleepiness and general health status. Sleep.

[10] Young T, Palta M, Dempsey J, Skatrud J, Weber S, Badr S (1993). The occurrence of sleep-disordered breathing among middle-aged adults. N Engl J Med.

[11] Duran J, Esnaola S, Rubio R, Iztueta A (2001). Obstructive sleep apnea-hypopnea and related clinical features in a population-based sample of subjects aged 30 to 70 yr. Am J Respir Crit Care Med.

[12] Bixler EO, Vgontzas AN, Lin HM, Calhoun SL, Vela-Bueno A, Kales A (2005). Excessive daytime sleepiness in a general population sample: the role of sleep apnea, age, obesity, diabetes, and depression. J Clin Endocrinol Metab.

[13] Stradling JR, Barbour C, Glennon J, Langford BA, Crosby JH (2000). Prevalence of sleepiness and its relation to autonomic evidence of arousals and increased inspiratory effort in a community based population of men and women. J Sleep Res.

[14] Stiefel F, Stagno D (2004). Management of insomnia in patients with chronic pain conditions. CNS Drugs.

[15] Young TB (2004). Epidemiology of daytime sleepiness: definitions, symptomatology, and prevalence. J Clin Psychiatry.

[16] Leger D, Poursain B, Neubauer D, Uchiyama M (2008). An international survey of sleeping problems in the general population. Curr Med Res Opin.

[17] Peres MF, Stiles MA, Siow HC, Silberstein SD (2005). Excessive daytime sleepiness in migraine patients. J Neurol Neurosurg Psychiatry.

[18] Barbanti P, Fabbrini G, Aurilia C, Vanacore N, Cruccu G (2007). A case–control study on excessive daytime sleepiness in episodic migraine. Cephalalgia.

[19] Seidel S, Hartl T, Weber M, Matterey S, Paul A, Riederer F, Gharabaghi M, Wober-Bingol C, Wober C (2009). Quality of sleep, fatigue and daytime sleepiness in migraine - a controlled study. Cephalalgia.

[20] Barbanti P, Aurilia C, Egeo G, Fofi L, Vanacore N (2013). A case–control study on excessive daytime sleepiness in chronic migraine. Sleep Med.

[21] Ødegård SS, Engstrøm M, Sand T, Stovner LJ, Zwart JA, Hagen K (2010). Associations between sleep disturbance and primary headaches: the third Nord-Trondelag Health Study. J Headache Pain.

[22] Kim J, Cho SJ, Kim WJ, Yang KI, Yun CH, Chu MK (2016). Excessive daytime sleepiness is associated with an exacerbation of migraine: A population-based study. J Headache Pain.

[23] Russell MB, Kristiansen HA, Saltyte-Benth J, Kvaerner KJ (2008). A cross-sectional population-based survey of migraine and headache in 21,177 Norwegians: the Akershus sleep apnea project. J Headache Pain.

[24] Society HCSotIH (2004). The International Classification of Headache Disorders: 2nd edition. Cephalalgia.

[25] Johns MW (1991). A new method for measuring daytime sleepiness: the Epworth sleepiness scale. Sleep.

[26] Johns MW (1994). Sleepiness in different situations measured by the Epworth Sleepiness Scale. Sleep.

[27] Beiske KK, Kjelsberg FN, Ruud EA, Stavem K (2009). Reliability and validity of a Norwegian version of the Epworth sleepiness scale. Sleep Breath.

[28] Whooley MA, Avins AL, Miranda J, Browner WS (1997). Case-finding instruments for depression. Two questions are as good as many. J Gen Intern Med.

[29] Pallesen S, Nordhus IH, Omvik S, Sivertsen B, Tell GS, Bjorvatn B (2007). Prevalence and risk factors of subjective sleepiness in the general adult population. Sleep.

[30] Vgontzas A, Cui L, Merikangas KR (2008). Are sleep difficulties associated with migraine attributable to anxiety and depression?. Headache.

[31] Ancoli-Israel S, Roth T (1999). Characteristics of insomnia in the united states: results of the 1991 national sleep foundation survey. I. Sleep.

[32] Johns MW, Hocking B (1997). Daytime sleepiness and sleep habits of Australian workers. Sleep.

[33] Gander PH, Marshall NS, Harris R, Reid P (2005). The Epworth sleepiness scale: influence of age, ethnicity, and socioeconomic deprivation. Epworth sleepiness scores of adults in New Zealand. Sleep.

[34] Sanford SD, Lichstein KL, Durrence HH, Riedel BW, Taylor DJ, Bush AJ (2006). The influence of age, gender, ethnicity, and insomnia on Epworth sleepiness scores: a normative US population. Sleep Med.

[35] Sander C, Hegerl U, Wirkner K, Walter N, Kocalevent RD, Petrowski K, Glaesmer H, Hinz A (2016). Normative values of the Epworth sleepiness scale (ESS), derived from a large German sample. Sleep Breath.

[36] Johns MW (1992). Reliability and factor analysis of the Epworth sleepiness scale. Sleep.

[37] Olson LG, Cole MF, Ambrogetti A (1998). Correlations among Epworth sleepiness scale scores, multiple sleep latency tests and psychological symptoms. J Sleep Res.

[38] Chervin RD, Aldrich MS (1999). The Epworth Sleepiness Scale may not reflect objective measures of sleepiness or sleep apnea. Neurology.

[39] Furuta H, Kaneda R, Kosaka K, Arai H, Sano J, Koshino Y (1999). Epworth sleepiness scale and sleep studies in patients with obstructive sleep apnea syndrome. Psychiatry Clin Neurosci.

[40] Rasmussen BK, Jensen R, Olesen J (1991). Questionnaire versus clinical interview in the diagnosis of headache. Headache.

